# Neural Indices of Emotion Regulatory Implementation Correlate With Behavioral Regulatory Selection: Proof-of-Concept Investigation

**DOI:** 10.3389/fnbeh.2022.835253

**Published:** 2022-04-28

**Authors:** Naomi B. Fine, Naama Schwartz, Talma Hendler, Tal Gonen, Gal Sheppes

**Affiliations:** ^1^Faculty of Social Sciences, School of Psychological Sciences, Tel Aviv University, Tel Aviv, Israel; ^2^Sagol Brain Institute Tel-Aviv, Tel Aviv Sourasky Medical Center, Tel Aviv, Israel; ^3^Sackler Faculty of Medicine, Tel Aviv University, Tel Aviv, Israel; ^4^Sagol School of Neuroscience, Tel Aviv University, Tel Aviv, Israel

**Keywords:** emotion regulation, fMRI, amygdala, distraction, reappraisal

## Abstract

“Do what you do best” conveys an intuition about the association between ability and preference. In the field of emotion regulation, ability and preference are manifested in two central stages, namely, implementation and selection of regulatory strategies, which to date have been mainly studied separately. Accordingly, the present proof-of-concept study wished to provide preliminary evidence for an association between neural indices of implementation ability and behavioral selection preferences. In this pilot study, participants performed a classic neuroimaging regulatory implementation task that examined their ability (neurally reflected in the degree of amygdala modulation) to execute two central regulatory strategies, namely, attentional distraction and cognitive reappraisal while viewing negative images. Then participants performed a separate, classic behavioral selection task that examined their choice preferences for using distraction and reappraisal while viewing negative images. Confirming our conceptual framework, we found that exclusively for distraction, which has been associated with robust amygdala modulation, a decrease in amygdala activity during implementation (i.e., enhanced ability) was associated with enhanced preference to behaviorally select distraction [*r*(15) = −0.69, *p* = 0.004]. These preliminary findings link between two central emotion regulatory stages, suggesting a clue of the adaptive association between neural ability and behavioral preference for particular regulatory strategies.

## Introduction

Conventional wisdom suggests that we tend to do what we do best. Whereas one person with high logical ability may be inclined to select to pursuit a degree in philosophy, another person with high coordination skills may select to chase a career in sports. Transcending conventional wisdom, it has been found that in fields such as philosophy (Motto, 1962) and sports (Macnamara et al., 2016), there is a strong relationship between ability and preference.

The conceptual association between ability and preference has been recently recognized in affective science, and in particular in emotion regulation (e.g., Silvers and Guassi Moreira, 2019 for a review). Within the currently dominant view of emotion regulation as composed of several key *interacting* stages (for reviews see Webb et al., 2012a; Bonanno and Burton, 2013; Gross, 2015; Sheppes et al., 2015; Sheppes, 2020), ability and preference are manifested in the two most studied regulatory stages, namely, in *implementation* and *selection*. Implementation refers to individuals’ ability to successfully modulate emotional responses *via* the execution of specific regulatory strategies, and selection refers to choices’ individuals make between strategies, that represent their regulatory preferences in different contexts. To date, despite the theoretical description of meaningful relationships between ability and preference and some conceptual arguments that link neural ability and behavioral preference during development (Silvers and Guassi Moreira, 2019), regulatory implementation and selection stages have been mostly studied separately (Sheppes, 2020). Multiple implementation studies increasingly emphasize the ability to modulate key neural affective responding systems *via* different regulatory strategies (e.g., Hajcak et al., 2010; Buhle et al., 2014; Etkin et al., 2015 for reviews). Emerging selection studies have convincingly described behavioral decision-making processes that constitute choice preferences for regulatory strategies in different contexts (Sheppes, 2014, 2020 for reviews).

As opposed to the multiple studies that investigated neural modulation of affective responding in regulatory implementation and behavioral selection separately, empirical studies linking between the two stages remain sparse and indirect. One type of indirect evidence (e.g., Kanske et al., 2012, 2015; Giuliani et al., 2014; Che et al., 2015; Paschke et al., 2016) comes from studies that measured the relationship between neural indices of strategy implementation and self-reported frequency of using different strategies (e.g., using the Emotion Regulation Questionnaire, Gross and John, 2003). While self-report questionnaires provide a general estimate of strategy use, they do not assess active behavioral selection between strategies in different contexts (see Sheppes, 2020 for discussion). A second type of indirect evidence comes from a single study that did the reverse: it measured behavioral regulatory selection but did not measure neural indices of strategy implementation (Doré et al., 2017). Specifically, this study evaluated neural indices of affective responding while participants naturally watched affective stimuli without regulating, could not assess regulatory implementation.

To fill these gaps, the present proof-of-concept small-scale study wished to provide preliminary evidence for the relationship between implementation ability as reflected by neural modulation of affective responding, and behavioral regulatory selection preferences. We rely on a central conceptual regulatory implementation and selection framework that focuses on two well-established cognitive regulatory strategies: distraction and reappraisal (see Sheppes and Gross, 2011; Sheppes, 2020, and also see, Ochsner and Gross, 2005; McRae, 2016 for reviews). Distraction that includes producing neutral thoughts, provides early attentional disengagement from emotional information processing before information undergoes elaborated meaning processing (Van Dillen and Koole, 2007). By contrast, reappraisal that includes forming neutral reinterpretations of emotional events involves attentional engagement with emotional information and appraising it affectively, prior to a change that occurs at a late semantic meaning stage (Sheppes and Gross, 2011).

According to our conceptual framework, the early attentional disengagement in distraction leads to stronger affective modulation relative to the late semantic meaning operation in reappraisal (Sheppes and Gross, 2011; Sheppes, 2020 for reviews). Supporting findings from multiple neuroimaging studies show that relative to reappraisal distraction results in more consistent, and robust modulation of a primary affective responding node—the *amygdala* (e.g., McRae et al., 2010; Kanske et al., 2011; Dörfel et al., 2014; Moodie et al., 2020, see also in the section “Results” complete replication of these findings in this study).

Given these converging findings, in the present proof-of-concept study we focused on amygdala modulation as the main target for assessing neural indices of regulatory implementation ability. Our focus on amygdala modulation is also congruent with the general notion that the amygdala is considered the most common target for assessing regulatory implementation outcomes (Ochsner et al., 2004; Etkin et al., 2015 for reviews). Moreover, many novel emotion regulation neurofeedback studies probe the modulation of the amygdala as the target for regulatory implementation (Bruhl et al., 2014; Paret et al., 2014) and link the extent of amygdala modulation during regulatory implementation with the improvement of emotion regulation skills (Herwig et al., 2019 for review).

The main goal of the present small-scale pilot study was to provide preliminary evidence for a possible association between neural implementation ability and behavioral selection preference. To that end, participants in this small-scale pilot study completed a classic fMRI regulatory implementation paradigm (c.f., Moodie et al., 2020), where amygdala modulation was assessed while participants were instructed to employ distraction or reappraisal while viewing negative images. Following the regulatory implementation paradigm, participants performed a classic regulatory selection paradigm (Sheppes et al., 2011), where they freely choose between distraction and reappraisal while viewing negative images.

Utilizing our conceptual framework, our hypothesis sought to examine whether a decrease in amygdala activity during implementation (i.e., enhanced ability) of a particular strategy is associated with enhanced behavioral selection preference of that strategy. Specifically, we tested the prediction that particularly for distraction, that has been associated with robust amygdala modulation (see also section “Results” for a replication), we found that a decrease in amygdala activity during implementation (i.e., enhanced ability) would be associated with enhanced preference to behaviorally select distraction. This prediction is also congruent with findings showing that healthy individuals are able to consider the costs and benefits associated with implementing distraction and reappraisal and adapt their behavioral regulatory selection preferences accordingly (Sheppes, 2020 for review).

## Materials and Methods

### Participants

In total, 21 healthy adults (11 males) ages 21–29 (mean age = 23.9, SD = 2.01) took part in our small-scale proof-of-concept pilot experiment and were paid 35 dollars for their participation. While clearly being a small study (see also later limitations), our sample size meets guidelines in the field regarding acceptable minimum sample sizes for pilot proof-of-concept studies (c.f., Mumford, 2012; Tashjian et al., 2018)^1^. Potential participants were recruited *via* electronic flyers from the campus community in Tel Aviv University, Israel. All the participants met the following inclusion criteria: (1) Right-hand dominance. (2) Hebrew as a native language. (3) fMRI compatibility (no embedded metal in body, not pregnant). (4) No reported history of mental disorders, and no current use of psychoactive medications. All participants had normal or corrected-to-normal vision were medically healthy and reported no history of serious head injury. All participants compiled the Code of Ethics of the World Medical Association—Helsinki Declaration and signed a written consent form of Tel-Aviv Sourasky Medical Center Ethics Committee. In total, four participants were excluded from analysis due to scanning artifacts in fMRI data caused by technical problems of the scanner, and two additional participants were excluded due to excessive movements of more than 3 mm head motion deviation that could not be accounted for using movement correction tools. Accordingly, the final sample for this pilot investigation included 15 participants (53% females, mean age = 25.4, SD = 2.02).

### Stimuli

In total, 278 normatively rated pictures including 256 negative pictures (valence: *M* = 2.617, SD = 0.467; arousal: *M* = 5.510, SD = 0.89), were used for the implementation and selection paradigms, and 22 additional neutral pictures (valence: *M* = 5.017, SD = 0.409; arousal: *M* = 2.892, SD = 0.600) were used in the implementation paradigm. Images were selected from the International Affective Picture System (IAPS; Lang et al., 2008), GAPED collection (GAPED; Dan-Glauser and Scherer, 2011), the Emotional Picture System (EmoPicS; Wessa et al., 2010) and the Stanford Psychophysiology Lab^2^.

### Procedure and Design

All the participants completed two paradigms in the MRI scanner: regulatory implementation, followed by regulatory selection. Prior to the scan, a practice phase of both tasks was conducted outside the scanner in order to ensure a full understanding of the tasks. In both tasks, stimulus presentation and data acquisition were controlled using E-prime software (Schneider et al., 2002).

#### Regulatory Implementation Task

The Regulatory Implementation Task was adopted from Moodie et al. (2020). This task included a practice phase in order to adequately learn and assure full comprehension of the different implementation conditions. Following, participants completed the main experimental phase in which they performed the implementation conditions.

Specifically, the paradigm included instructing participants to implement four different conditions, two regulation conditions, namely, “distraction” and “reappraisal”; and two control conditions of passively viewing images without emotion regulation, namely, “watch,” for negative images and “view” for neutral images.

“Distraction” instructions required participants to disengage attention from the presented picture by producing unrelated neutral thoughts (i.e., visualizing geometric shapes or daily activities). “Reappraisal” instructions involved engaging with the emotional picture and changing its emotional meaning by thinking of a better outcome or focusing on an aspect of the situation that is less negative (c.f., Thiruchselvam et al., 2011; Sheppes, 2014; Shafir et al., 2015). The instructions of “View” and “Watch” involved looking at the picture and experiencing natural thoughts and feelings as they arise, with the sole difference being that “Watch” was coupled with a negative picture while “View” was always followed by a neutral picture (c.f., Thiruchselvam et al., 2011)^3^.

##### Practice Phase

Participants learned (six trials) and then practiced (six trials) how to implement the four different conditions: “distraction” and “reappraisal” (two trials each), “watch” and “view” (one trial each). During this phase participants were asked to verbalize their responses, to ensure they comprehended and implemented the instructions correctly. Following practice outside the scanner and before performing the task, individuals performed eight additional practice trials inside the scanner.

##### Experimental Phase

The actual task consisted of 162 trials, divided into six blocks of equal length. Each block contained 27 trials such as 16 implementation (distract OR reappraise) trials, eight “watch” (negative) trials, and three “view” (neutral) trials. In an effort to prevent participants from confusing or combining implementation of the two regulation strategies (c.f., Thiruchselvam et al., 2011), only a single strategy (distraction or reappraisal) was included in a particular block. Stimuli were organized in a pseudorandom order within each block, randomly assigned to regulatory instructions, and order of blocks was counterbalanced across participants. This design resulted in obtaining 48 trials for each of the three instruction type independent variable conditions (Distraction, Reappraisal, Watch).

**FIGURE 1:**
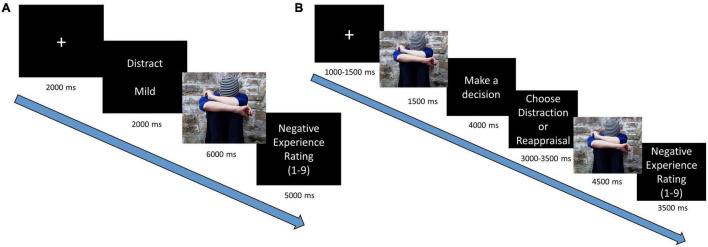
Trial sequence in emotion regulation paradigms: **(A)** neural implementation task and **(B)** behavioral regulatory selection task.

The trial sequence (Figure 1A) began with a fixation cross (two seconds), followed by a cue screen (two seconds), which informed about the regulatory instructions (“Watch,” “Distraction,” “Reappraisal,” “View”) and the emotional intensity of the upcoming stimuli (“Mild” or “Intense,” “Neutral”). Then a pictorial stimulus was displayed on the entire screen (six seconds) while participants were asked to implement the requested instruction for the total duration. Following the picture presentation, participants were asked to rate their level of negative experience on a Likert scale that was presented for five seconds (1 = “not negative at all,” 9 = “very negative”) (For rational and analyses of self-report measures see Supplementary Appendix A).

#### Regulatory Selection Task

The regulatory selection task was adopted from Sheppes et al. (2011). This task included a practice phase in order to adequately learn and assure full comprehension of the different selection conditions. Following, participants completed the main experimental phase in which they selected their preferred strategy between regulatory conditions. Specifically, the paradigm included selecting between reappraisal and distraction while viewing negative images. Instructions for how to employ distraction and reappraisal were identical to the regulatory implementation task.

##### Practice Phase

Participants practiced (two trials) how to behaviorally select between distraction and reappraisal, by choosing for each trial the strategy that they think would most help them feel less negative toward the picture.

##### Experimental Phase

The actual task consisted of 90 trials, divided into three blocks of equal lengths. Each block contained 30 trials (15 high and 15 low emotional intensity pictures—see Supplementary Appendix C for secondary analyses) and stimuli were organized in a pseudorandom order within each block.

The trial sequence (Figure 1B) began with a fixation cross (for 1,000/1,250/1,500 ms jittered across trials), followed by a brief presentation of a pictorial stimulus (one second). Then participants viewed a screen, which asked them to select between “Reappraisal” or “Distraction” (four seconds), following which two buttons appeared, allowing participants to execute their preferred regulatory choice (3,000/3,250/3,500 ms).

Assignment of the strategies to the response buttons was randomized across trials (c.f., Shafir et al., 2016). After pressing their chosen strategy, the pictorial stimulus reappeared (four and a half seconds), and the participants were asked to implement the strategy they chose. Each trial ended with a Likert scale as described above, presented for three and a half seconds^4^. Regulatory selection preference was calculated by the percentage of choosing distraction (over reappraisal).

### MRI Data Acquisition and Preprocessing

The 3T scanner (GE Signa LX Horizon Echospeed) located in the Wohl Institute for Advanced Imaging, in Tel Aviv Sourasky Medical Center was used to acquire functional MRI data. Each scan consisted of thirty-two axial slices (3.5 mm thick each, with no gap between slices) acquired by TE = 35 ms, TR = 2,500 ms, flip angle = 90, field of view = 22 cm, matrix: 128 × 128. The anatomical scan consisted of twenty-seven brain slices with 1 × 1 × 1 mm resolution and 256 × 256 matrix. Structural scans included a T1-weighted 3D axial spoiled gradient echo (SPGR) pulse sequence (TR/TE = 7.92/2.98 ms, flip-angle = 15°, pixel-size = 1 mm, FOV = 256 mm × 256 mm, slice-thickness = 1 mm). Responses in MRI were recorded with both right and left index fingers using two buttons on a four-button response box. Pre-processing was performed using BrainVoyager 2.1.4 (Brain Innovation, Maastricht, Netherlands: Goebel et al., 2006). Each of the participant’s sequential functional volumes was realigned to the first scan and co-registered to its anatomical MRI. Slice time correction with cubic spline interpolation, was performed in an ascending interleaved fashion. Motion detection with three translation and three rotations parameters was used to align positioning to the first volume. High pass temporal filtering was conducted for each individual voxel separately using a linear-trend removal with a high-pass filter of 0.008 Hz. Additionally, images were spatially smoothed with a 6-mm FWHM kernel. Anatomical data was translated and rotated into AC–PC plane, then normalized and standardized into Talairach space.

### fMRI Data Analysis

#### Regulatory Implementation Task

For the regulatory implementation task, we conducted the following procedures: at the first level, single-subject analysis, blood oxygen level dependent (BOLD) signal during the implementation phase were normalized and entered to a General Linear Model (GLM) for each subject separately. These data were modeled and convolved with a canonical hemodynamic response function. Movement parameters, white matter and ventricles mean signal were added as covariates of confounds. A second-level, multi-subject random effects (RFX) GLM was computed to create contrasts of interest.

#### Contrasts of Interest

In order to extract differential amygdala modulation associated with implementing distraction and reappraisal, we created two contrasts of interest using a statistical threshold of *p* < 0.05, FDR corrected (c.f., empirical papers using identical gold-standard contrasts in implementation paradigms, Ochsner et al., 2002; McRae et al., 2010; Moodie et al., 2020). We first subtracted the *Watch* condition from each regulatory condition, so that the chosen contrasts (Distraction > Watch, Reappraisal > Watch) exclude general neural activations that are elicited by viewing a pictorial stimulus without regulating (e.g., sensory, visual, and emotion generation activations) from activations associated with emotion regulatory implementation. In the second step, the analysis conducted a *t*-test between the two regulatory conditions in the following matter: it employs a GLM involving the subtraction of two contrasts [e.g., (Distraction > Watch) > (Reappraisal > Watch), or (Reappraisal > Watch) > (Distraction > Watch)] in order to capture activations that are strategy *specific* [i.e., activations that are specific for distraction beyond reappraisal in the (Distraction > Watch) > (Reappraisal > Watch) contrast].

#### Amygdala ROI

**FIGURE 2:**
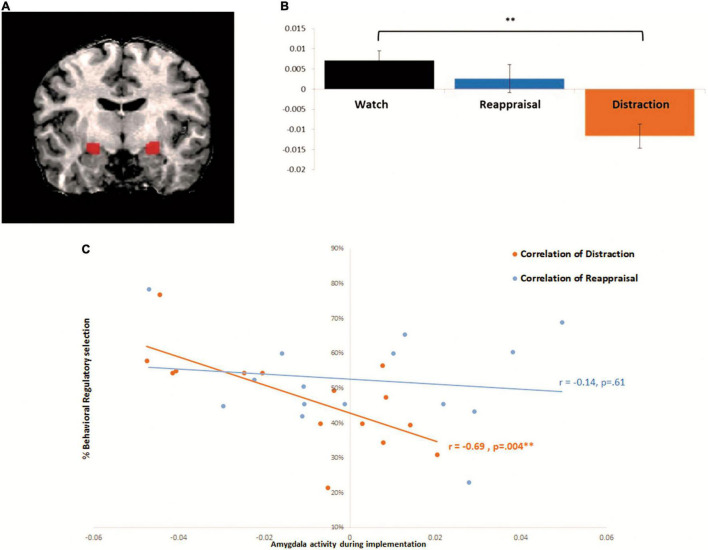
Amygdala activity during implementation correlates with behavioral selection during distraction but not reappraisal: **(A)** bilateral amygdala functional ROI: active voxels during both Watch > Distraction and Reappraisal (which reflect the reduction of amygdala activity by both ER strategies relative to Watch) were turned into a mask to define the functional region of interest (right: 21 −12 −8, left: −23 −12 −7). **(B)** Beta parameter estimates from the bilateral amygdala (extracted from functional ROI as described in panel **A**). Repeated measures ANOVA of Instruction: (Watch, Reappraisal, Distraction) *F*(2, 28) = 4.837, *p* = 0.015, and showed significant difference between distraction and watch *F*(1,14) = 17.73, *p* = 0.0008. **(C)** Correlation between amygdala activity and behavioral selection: In orange—correlation between amygdala activity during implementation and behavioral selection of Distraction [Pearson correlation *r*(15) = −0.6915, *p* < 0.005, Bonferroni corrected for multiple comparisons]. In blue—correlation between amygdala activity during implementation and behavioral selection of Reappraisal *r*(15) = −0.14, *p* = 0.6. *p < 0.05, **p < 0.005, ***p < 0.0005. Error bars represent standard error of mean.

According to our *a priori* amygdala hypothesis, we combined anatomical and functional approaches for the analyses as follows (e.g., Ben-Simon et al., 2015; Jensen et al., 2021). First, we localized a mask of the amygdala using a conventional anatomical brain atlas [Talairach brain atlas accompanying the BrainVoyager QX software package (BVQX 2.4; Brain Innovation)]. Second, because our hypothesis regarded differential functional activation in the amygdala under two strategies, within the anatomical defined mask, we conducted functional contrast of interest (Distraction > Watch and Reappraisal > Watch, as described above) in order to capture activations that are strategy specific. Main correlation analysis included significant voxels from this combined procedure (Talairach coordinations right: 21 -12 -8, left: -23 -12 -7). Laterality was tested using repeated measures ANOVA, with no difference between left and right amygdala across regulatory strategies [*F*(1,14) = 4.324, *p* = 0.08], and so bilateral ROI was used for further analyses. Laterality was tested using repeated measures ANOVA, with no difference between left and right amygdala across regulatory strategies [*F*(1,14) = 4.324, *p* = 0.08], and so bilateral ROI was used for further analyses (Figure 2A). In all analyses, brain activations were assessed at a statistical threshold of *p* < 0.05 using FDR correction.

## Results

Secondary behavioral findings in the regulatory implementation task and their association to preferences in selection task are reported in the Supplementary Appendix A.

### Regulatory Selection Task—Behavioral Preference

To test the regulatory selection preference, percentage of choosing distraction (over reappraisal) was calculated. Across intensities, distraction was preferred in 45% of the trials (SD = 1.46).

### Neural Indices

#### Amygdala Modulation During Implementation

According to our conceptual framework (Sheppes and Gross, 2011; Sheppes, 2020 for reviews), and previous neural findings (e.g., McRae et al., 2010; Moodie et al., 2020) the early attentional disengagement in distraction, leads to stronger amygdala modulation relative to the late semantic meaning operation in reappraisal (Dörfel et al., 2014). To test this premise we examined amygdala modulation in distraction and reappraisal relative to watch. Accordingly, beta parameter estimates during the implementation task were extracted from bilateral amygdala and were subjected to one-way repeated-measure ANOVA, with regulatory instructions (Reappraise/Distract/Watch) as the independent variable.

Congruent with previous findings, a significant main effect for regulatory instruction was found, *F*(2, 28) = 4.83, *p* = 0.015, η*_*p*_*^2^ = 0.256, with follow up comparisons indicating that relative to watch, mean β = 0.007, SD = 0.01, 95% CI = −0.002, 0.017, during distraction, mean β = −0.01, SD = 0.02, 95% CI = −0.02, 0.001, there was a significant decrease in amygdala activity, *F*(1,14) = 17.73, *p* < 0.001, η*_*p*_*^2^ = 0.56, 95% CI = −0.03, -0.003. By contrast, reappraisal, mean β = 0.002, SD = 0.02, 95% CI = −0.01, 0.01, did not yield a significant decrease in amygdala activity relative to watch, *F*(1,14) = 0.48, *p* = 0.49, η*_*p*_*^2^ = 0.14, 95% CI = −0.02, 0.01 (see Figure 2B)^5^.

### Ability and Preference

Is the decrease of amygdala activity during distraction implementation associated with enhanced preference to behaviorally select distraction?

To test our hypothesis, we measured the Pearson correlation between neural amygdala activity during distraction in the regulatory implementation paradigm, and the behavioral proportion of distraction choice during the regulatory selection paradigm (across intensities). Supporting our prediction, we found that for distraction, that was associated with robust amygdala modulation, the decrease of amygdala activity during implementation was associated with enhanced preference to behaviorally select distraction, *r*(15) = −0.69, *p* = 0.004 (Bonferroni corrected) (see Figure 2C); and that was not the case for reappraisal, *r*(15) = −0.14, *p* = 0.61.

To examine the stability of this correlation we employed a non-parametric bootstrap procedure for CIs (Barber and Thompson, 2000; Carpenter and Bithell, 2000), which is the suitable method for small samples (c.f., Briggs et al., 1997; Preacher and Hayes, 2008; Dwivedi et al., 2017). Specifically, each variable was bootstrapped 10,000 times, followed by computing a correlation between the two variables. This repeated procedure yielded an average correlation value and averaged confidence interval that estimates the margin of error and whether it overlaps with the value zero which denotes no effect. The result of this analysis showed that the confidence interval did not include the value zero whether we assumed that the distribution of the sample is normal [95% CI (−1.24, −0.17) or not 95% CI (−1.94, −0.91)].

To provide further support for the differential association between amygdala modulation during implementation and behavioral selection of distraction and reappraisal, we tested the difference between these two dependent correlations, with the percentage of behavioral preference as a common variable. To that end, we first converted each correlation coefficient into a *z*-score using Fisher’s *r*-to-*z* transformation and then computed the covariance of estimates to use it in the asymptotic *z* test (Lee and Preacher, 2013). Supporting our prediction, we found a significant difference between the significant distraction correlation, and the non-significant reappraisal correlation, *Z*(1,14) = −2.06, *p* = 0.01.

## Discussion

While lay intuition and sound conceptual accounts predict that individuals tend to do what they do best, to date, the empirical evidence linking ability and preference in the context of emotion regulation remains sparse and indirect. Providing preliminary evidence in a small-scale study we found support for an association between neural regulatory implementation ability and behavioral regulatory selection preferences. Specifically, supporting our prediction we found that exclusively for distraction, which was associated with robust amygdala modulation, a decrease in amygdala activity during implementation was associated with enhanced preference to behaviorally select distraction and this was further supported by internal stability of the correlation. As expected, this relation was not found for reappraisal.

In this study relative to a watch condition, distraction was associated with strong amygdala modulation, but reappraisal was not. Prior studies indeed show that relative to reappraisal, distraction results in more consistent, and robust modulation of the amygdala (e.g., McRae et al., 2010; Kanske et al., 2011; Dörfel et al., 2014; Moodie et al., 2020). The lack of amygdala modulation in reappraisal is consistent with several prior studies showing no (Sang and Hamann, 2007; Silvers et al., 2015) or inconsistent (Dörfel et al., 2014) amygdala modulation. While a prior meta-analysis (Buhle et al., 2014) found that across studies reappraisal is generally associated with amygdala modulation, reappraisal is a heterogeneous category (see McRae et al., 2012) that includes different tactics (Moodie et al., 2020). It might be of further interest to examine whether reappraisal tactics that involve engagement with emotional processing show lower amygdala modulation compared to other reappraisal tactics (such as distancing and reality challenge) that are considered more disengaging.

Our small-scale proof-of-concept study showing an association between neural implementation ability and behavioral selection preferences, extends prior research that tended to study each regulatory stage separately (see Sheppes, 2020 for review). Our preliminary findings provide initial empirical support to recent conceptual accounts that emphasize the importance of the interaction between regulatory stages and particularly the link between implementation and selection (e.g., Silvers and Guassi Moreira, 2019).

Our findings showed that for distraction that successfully modulates the amygdala, higher neural implementation ability (lower amygdala activity) was associated with higher behavioral distraction selection preference. This preliminary finding contributes to the extensive research on regulatory implementation in showing that successive feedback loops between successful distraction implementation and increased preference can facilitate an adaptive regulatory profile across time. The association between implementation and selection also contributes to the growing body of research on selection. Our findings suggest a neural component that represents individuals’ ability to regulate as a predictor of regulatory selection, thus adding an important determinant to the established affective, cognitive, and motivational determinants of regulatory selection (Sheppes, 2020).

Demonstrating an adaptive profile in healthy individuals that links increased ability with enhanced preference, has several clinical implications. Emotion dysregulation and psychopathology can be characterized by a dissociation between ability and preference. One form of dysregulation can involve preferring strategies one has impaired ability to implement, and a second form of dysregulation may involve not selecting strategies one has adequate ability to implement. Applying these insights to treatment, clinical interventions can transcend their focus on improving ability (e.g., Herwig et al., 2019) or regulatory preferences (e.g., Enav et al., 2019; Bahrami et al., 2020) to strengthen links between ability and selection preference.

Despite the novel features of the study, it is important to mention several limitations and future directions. First and foremost our results should be interpreted as preliminary as this was a small-scale proof-of-concept study. Despite this clear limitation and modest conclusions that can be derived from pilot studies, it is important to mention that the present hypotheses and supporting findings are derived from our well-established framework (Sheppes and Gross, 2011; Sheppes, 2020). Importantly, this study provides direct replication of prior neural implementation (e.g., Moodie et al., 2020; see also supporting findings by McRae et al., 2010; Kanske et al., 2011; Dörfel et al., 2014) and behavioral selection findings (Sheppes, 2014, 2020). In addition, the primary correlational analysis reported was conducted using the simplest single-level analysis that took into consideration the limited sample size of the study and power which yield a recommendation to perform the most conservative and basic analysis (see Everitt and Howell, 2021) and internal reliability of the primary correlation was demonstrated. Despite the replication of prior findings and stability of current findings that increase our trust in the present findings, it is crucial that future studies would replicate our results in a larger sample.

Second, this study utilized a well-established paradigm in which regulatory implementation is experimentally manipulated by contrasting conditions that allow extracting the regulation of emotion (e.g., distraction) from the generation of emotion (e.g., watch) in an experimental paradigm that is known to interpret amygdala modulation as representing regulatory ability (Ochsner et al., 2004; Ochsner and Gross, 2014; Etkin et al., 2015). This strengthens the validity of amygdala activity as representing regulatory ability and weakens arguments of reverse inference from neural activation to psychological functions. Nevertheless, the amygdala (and all other brain regions) is involved in several psychological functions and therefore one should be cautious in inferring a particular psychological function from the observed activity of a particular brain region.

Third, while distraction has clear benefits it also has significant costs, since distraction does not entail the processing of emotional events. Therefore, making sense of emotional events does not occur, which is non-beneficial for long-term adaptation (Wilson and Gilbert, 2008). Despite clear costs, it is important to view distraction as a valid regulatory option that also has significant benefits. In general, there is a growing theoretical consensus that regulatory strategies are not inherently “good” or “bad”; rather, regulatory strategies differ in their consequences in varying contexts (Sheppes, 2020). Specifically, distraction has clear benefits in the short run when facing highly intense emotional situations since distraction’s early attentional disengagement operation attenuates negative emotions quickly and effectively, with relatively minimal effort (e.g., Sheppes and Meiran, 2007, 2008; Sheppes and Gross, 2011; Shafir et al., 2015).

In summary, although this study investigated the two most established regulatory engagement and disengagement strategies (Sheppes, 2020), future studies may consider examining whether the association between ability and preference is stable across time as well as testing other strategies along the engagement disengagement continuum (e.g., Moodie et al., 2020).

## Data Availability Statement

The original contributions presented in the study are included in the article/Supplementary Material, further inquiries can be directed to the corresponding authors.

## Ethics Statement

The study was approved by the Tel Aviv Sourasky Medical Center Ethics Committee (No. 06-292). The patients/participants provided their written informed consent to participate in this study.

## Author Contributions

NS and TG designed and performed the experiments and analyzed the data. NF wrote the manuscript with support from GS and TG. TH and GS conceptualized and supervised all aspects of the study. All authors contributed to the article and approved the submitted version.

## Conflict of Interest

The authors declare that the research was conducted in the absence of any commercial or financial relationships that could be construed as a potential conflict of interest.

## Publisher’s Note

All claims expressed in this article are solely those of the authors and do not necessarily represent those of their affiliated organizations, or those of the publisher, the editors and the reviewers. Any product that may be evaluated in this article, or claim that may be made by its manufacturer, is not guaranteed or endorsed by the publisher.

## Footnotes

1 Although we rely on two separate paradigms that yield large effect sizes (see Webb et al., 2012b; Buhle et al., 2014; Picó-Pérez et al., 2017 for meta-analyses of regulatory implementation studies, reporting an average effect size of *r* = 0.41; and see Matthews et al., 2021 a meta-analysis of regulatory selection studies, reporting an average effect size of *r* = 0.67), we were unable to compute an *a priori* power analysis using prior effect size since there is no study that investigated the association between neural implementation and behavioral selection preferences.

2 In addition to our central hypothesis, a secondary hypothesis assessed the role of emotional intensity. Accordingly, half of the negative images were of high intensity and the other half of low intensity. See Supplementary Appendix C for full details and analyses.

3 We included the view condition (neutral images) in only a small fraction of trials (11%) primarily to prevent predictability and habituation to negative pictures while keeping participants engaged and concentrated in the task. For this reason, we did not analyze nor discuss this condition further (c.f., Ochsner et al., 2002).

4 It has been widely documented (Scheibe et al., 2015; Sheppes, 2020 for review) that the regulatory selection paradigm is not designed, and thus cannot provide, accurate assessment of the affective consequences of regulatory selection. This is because inferences about differential efficacy of regulatory strategies following regulatory selection (e.g., whether in a high-intensity condition distraction regulatory decisions result in lower negative affect relative to reappraisal regulatory decisions) require equating stimuli’s pre-choice emotional intensity for each of the two conditions (e.g., equating the initial negativity of stimuli that led to distraction relative to reappraisal regulatory decisions). In the regulatory selection paradigm, experimental matching of intensity is not possible because participants freely select between strategies for each stimulus. Accordingly, since post-choice ratings are un-interpretable we made an *a priori* decision to not analyze them. Note that post-choice ratings are typically included in the paradigm to remind participants the goal of regulatory selection, which is to choose on each trial the regulatory option they think will assist them the most to reduce their negative emotional experience.

5 For further whole-brain analyses replicating the amygdala ROI analyses and replicating prior studies showing differential recruitment of attentional and semantic meaning neural networks see Supplementary Appendix B.
