# Supplementary material for: Neural Indices of Emotion Regulatory Implementation Correlate With Behavioral Regulatory Selection: Proof-of-Concept Investigation
Source: Front Behav Neurosci. 2022 Apr 28;16:835253. doi: 10.3389/fnbeh.2022.835253 (PMC9096347; doi:10.3389/fnbeh.2022.835253)
Supplement: Supplementary file 1 [file Table_1.docx]

Supplementary Material

**Appendix A- Secondary behavioral findings in the Regulatory Implementation and Selection Tasks**

*1. Regulatory Implementation Task - Self-report Affective Ratings:*

To test the efficacy of reappraisal and distraction in reducing negative experience, self-report ratings were entered to a repeated measures one-way ANOVA of Regulatory Instructions (Watch, Reappraisal, Distraction) which revealed significant main effect, F(2, 40)=7.81, *p*=0.001, η*_p_*^2^= 0.28. Corresponding with previous findings, reappraisal and distraction were equally effective in reducing negative experience, showing heightened negative affect in watch (*M=*5.23, *SD=*0.63) compared to reappraisal, (*M=*4.89, *SD=*0.7), F(1,20)=10.91, *p*=0.003, η*_p_*^2^ = 0.22 and to distraction, (*M=*4.9, *SD=*0.74), F(1,20)=8.06, *p*=0.010,η*_p_*^2^ =0.29.

2. *Ability and Preference- Regulatory Implementation and Selection Task:*

Self-report measures are prone to multiple biases (Arnold & Feldman, 1981; Howard & Dailey, 1979; Poole et al., 2017), and across multiple domains it has been repeatedly found that individuals report inaccurate estimations of their abilities (in domains other than emotion regulation see for example Campbell, 1986; Freund & Kasten, 2012 and in emotion regulation see for example Pierce et al., 2021). These self-report biases have been formally described in the well-known Dunning-Kruger phenomenon showing that individuals with low objective ability tend to overestimate it in their self-reports (Dunning, 2011; Kruger & Dunning, 1999) including in self-reports of of emotion regulation ability (Sheldon et al., 2014)). Nevertheless, we report the non-significant associations between self-reported ratings of distraction in Implementation Task and preference of distraction in Regulatory Selection Task (r(15)= -0.29, *p*=0.29).

**Appendix B - Validation of neural networks associated with distraction and reappraisal implementation**

**Methods**

In order to replicate prior findings and demonstrate differential neural networks associated with implementing distraction and reappraisal, we created two contrasts of interest using statistical threshold of p < 0.05 and FDR correction. To examine if the implementation of distraction recruits brain regions associated with attention control, we used the contrast (Distraction> Watch). To examine if the implementation of reappraisal recruits brain regions associated with semantic meaning, we used the contrast (Reappraisal > Watch). Since we wanted to identify unique regulative activity by distraction (attention) and reappraisal (semantic meaning), without sensory or emotion generation we subtracted the Watch condition from both contrasts. Then we conducted a t-test between neural activity elicited in the distraction contrast (Distraction>Watch) relative to the reappraisal contrast (Reappraisal>Watch) to find unique activations by distraction, and compared (Reappraisal >Watch) > (Distraction> Watch) to find regions that are active only in reappraisal but not in distraction and watch.

**Results**

Replicating prior findings and supporting our framework, increased activity during distraction > watch (relative to reappraisal> watch) was found in dorsal regions such as bilateral Inferior Parietal Lobule (IPL), dorsolateral prefrontal cortex (DLPFC) and dorsal posterior cingulate cortex (dACC), which are closely associated with attention control (Kanske et al., 2011, See Fig. S1 and Table S1). By contrast, increased activity during reappraisal > watch (relative to distraction > watch) was found in ventral activations in prefrontal and inferotemporal regions, which are associated with semantic meaning and interpretation (Badre & Wagner, 2007; Ochsner et al., 2012; Price & Drevets, 2010). Also, bilateral amygdala and ventral occipital cortex were found to be more active during reappraisal than distraction, which have been previously related to processing affectively arousing stimuli and modulating their encoding into memory (LeDoux, 2000; Phelps, 2006; Sabatinelli et al., 2005).


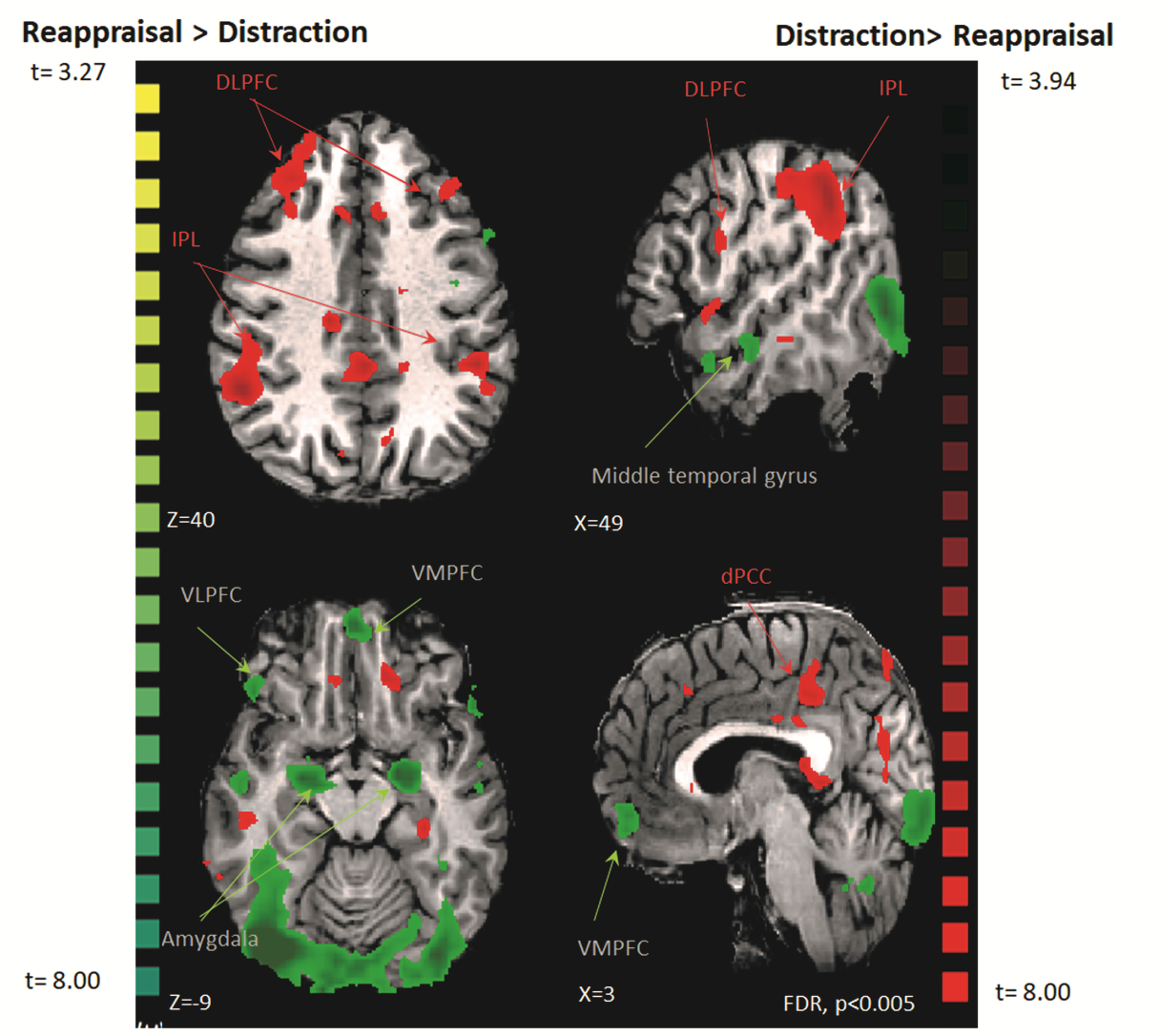

Figure S1. Distraction and Reappraisal Evoked dorsal vs. ventral regulation networks: The images present the results of a t- test between the contrast distraction> watch vs. the contrast reappraisal > watch, N=15, p(FDR)<.005. In red, increased activity during distraction > watch (relative to reappraisal> watch) was found in bilateral Inferior Parietal Lobule (IPL), dorsolateral prefrontal cortex (DLPFC) and dorsal posterior cingulate cortex (dACC). In blue, increased activity during reappraisal > watch (compared to distraction > watch) was found in bilateral amygdala, right ventrolateral prefrontal cortex (VLPFC), right ventromedial prefrontal cortex (VMPFC) and middle temporal gyrus.

Table S1. Distinct regulative networks for Distraction and reappraisal

**

**Appendix C- Ability and Preference:** **Is the decrease of amygdala activity during distraction implementation associated with enhanced preference to behaviorally select distraction in high intensity?**

In addition to our main hypothesis, we sought to assess whether the association between neural implementation ability and behavioral selection preferences would be particularly evident for high intensity stimuli. Specifically, according to our framework differences between distraction and reappraisal implementation are particularly expected in high intensity situations (Shafir et al., 2015; Sheppes & Meiran, 2007), and the behavioral selection preference for distraction is particularly evident in high intensity situations (Sheppes et al., 2011, 2014). Accordingly, we examined whether the expected relationship between the degree of amygdala modulation during distraction implementation would be associated with enhanced preference to behaviorally select distraction, particularly in high intensity situations. Note that this prediction is described as secondary, because there is only one neuroimaging study that investigated the differences between distraction and reappraisal across different intensities (Moodie et al., 2020) and because our study sample size limits our ability to meticulously investigate the moderating role of intensity.

**Methods**

**Stimuli**

Images included 128 high intensity (valence: M = 2.07, SD = 0.43; arousal: M = 6.13, SD = 0.8) and 128 low intensity negative pictures (valence: M =3.15, SD =0.5; arousal: M =4.78, SD =1.01), significantly differing in normative ratings of valence and arousal (both F's >49, *p*'s < .00001). This categorization was proved in prior studies to be useful in creating differential regulatory patterns in implementation (Shafir et al., 2015) and selection (Sheppes et al., 2011, 2014)^[[1]](#footnote-1)^. Stimuli were organized in a pseudorandom order within each block, randomly assigned to regulatory instructions so there were no more than three consecutive trials with the same emotional intensity and instruction (c.f., Sheppes, 2014; Sheppes et al., 2011).

**Results**

To test the regulatory selection preference, percentage of choosing distraction (over reappraisal) was calculated. Corresponding with previous findings, preferred strategy for regulation of high intensity pictures was distraction (M=60%, SD=1.87); opposed to low intensity trials in which distraction was preferred only 31% of the trials (M=31%, SD=1.7).

In order to test our secondary hypothesis whether the degree of amygdala activity during distraction implementation is associated with a preference to behaviorally select distraction in high intensity situations, we conducted the following steps: For each participant, beta parameter estimates of distraction were extracted from amygdala ROI during implementation task and subjected into a Pearson correlation with the percentage of choosing distraction (over reappraisal) in the subsequent choice task. Consistent with our secondary hypothesis, in high intensity situations, amygdala activity during distraction in the implementation task, was found to be significantly correlated with the percentage of choosing distraction in high intensity trials on the regulatory selection task, r(15)=-0.66, *p*=0.007 (FDR corrected for multiple comparisons). By contrast and as expected, amygdala activity during reappraisal implementation in high intensity was not correlated with the percent of choosing reappraisal in high intensity in the regulatory selection task, r(15)=0.07, *p*=0.79 (See Fig. S2). To provide further support for the differential association between amygdala modulation during implementation and behavioral selection of distraction but not reappraisal, we tested the difference between these two dependent correlations, with the percentage of behavioral preference as a common variable. To that end, we first converted each correlation coefficient into a z-score using Fisher's r-to-z transformation and then computed the covariance of estimates to use it in asymptotic z test (Lee & Preacher, 2013). Supporting our predictions, we found a significant difference between the significant distraction correlation and the non-significant reappraisal correlation, Z(1,14)= -2.45, *p*<0.01.

As opposed to the expected pattern in high intensity, in low intensity situations, amygdala activity during distraction in the implementation task, was not significantly correlated to the percentage of choosing distraction in high intensity trials on the subsequent selection task, r(15)=-0.52, *p*=0.18 (FDR corrected for multiple comparisons).

***Low Emotional intensity: A) Planned comparisons of 3*2 repeated measures ANOVA on parameter beta estimates extracted from functional ROI, indicated that reappraisal, mean beta=0.003, SD=0.02, 95% CI= -0.012,0.0184, did not decrease amygdala activity relative to watch, mean beta=0.0007, SD=0.01, 95% CI= -0.008,0.01, F(1,15)= 0.223, *p*= 0.644, η*_p_*^2^=0.015. However, distraction, mean beta=-0.018, SD=0.022, 95% CI= -0.03,-0.005, effectively decreased amygdala activity relative to watch, F(1,15)=19.945, *p*= 0.00053, η*_p_*^2^=0.587. B) We did not expect and did not find significant correlation between amygdala activity during implementation of reappraisal and preference to choose reappraisal in low intensity trials on a subsequent choice task, r(15)=-0.0006, *p*=0.98.


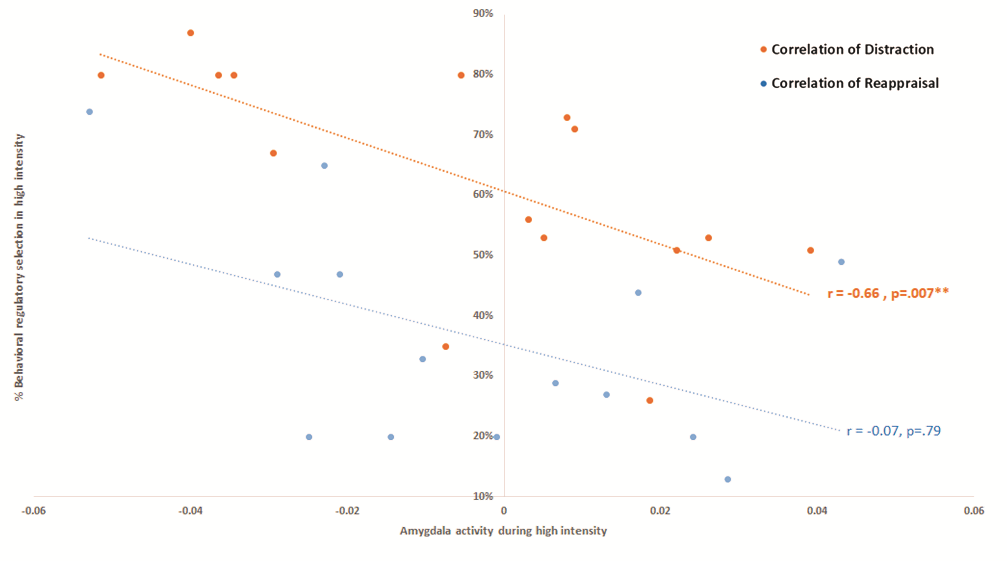


Figure S2. Amygdala activity during distraction (not reappraisal) correlates with its behavioral selection, in high intensity trials: In orange- correlation between amygdala activity during implementation and behavioral selection of Distraction in high intensity trials, Pearson correlation r(15)=-0.66,*p*=0.007, (Bonferroni corrected for multiple comparisons). In blue- correlation between amygdala activity during implementation and behavioral selection of Reappraisal in high intensity trials- r(15)=-0.07 ,*p*=0.79.

**References**

Arnold, H. J., & Feldman, D. C. (1981). Social Desirability Response Bias in Self-Report Choice Situations. *Academy of Management Journal*. https://doi.org/10.2307/255848

Badre, D., & Wagner, A. D. (2007). Left ventrolateral prefrontal cortex and the cognitive control of memory. In *Neuropsychologia*. https://doi.org/10.1016/j.neuropsychologia.2007.06.015

Campbell, J. D. (1986). Similarity and Uniqueness: The Effects of Attribute Type, Relevance, and Individual Differences in Self-Esteem and Depression. In *Journal of Personality and Social Psychology* (Vol. 50, Issue 2). https://doi.org/10.1037/0022-3514.50.2.281

Dunning, D. (2011). The Dunning – Kruger Effect : On Being Ignorant of One ’ s Own Ignorance. In *Advances in Experimental Social Psychology* (1st ed., Vol. 44). Elsevier Inc. https://doi.org/10.1016/B978-0-12-385522-0.00005-6

Freund, P. A., & Kasten, N. (2012). How smart do you think you are? A meta-analysis on the validity of self-estimates of cognitive ability. *Psychological Bulletin*, *138*(2), 296–321. https://doi.org/10.1037/a0026556

Howard, G. S., & Dailey, P. R. (1979). Response-shift bias: A source of contamination of self-report measures. *Journal of Applied Psychology*. https://doi.org/10.1037/0021-9010.64.2.144

Kanske, P., Heissler, J., Schönfelder, S., Bongers, A., & Wessa, M. (2011). How to Regulate Emotion? Neural Networks for Reappraisal and Distraction. *Cerebral Cortex*, *21*(6), 1379–1388. https://doi.org/10.1093/cercor/bhq216

Kruger, J., & Dunning, D. (1999). Unskilled and Unaware of It : How Difficulties in Recognizing One ’ s Own Incompetence Lead to Inflated Self-Assessments. *Journal of Personality and Social Psychology*, *77*(6). https://doi.org/10.1037/0022-3514.77.6.1121

LeDoux, J. E. (2000). Emotion Circuits in the Brain. *Annual Review of Neuroscience*. https://doi.org/10.1146/annurev.neuro.23.1.155

Lee, I. A., & Preacher, K. J. (2013). Calculation for the test of the difference between two dependent correlations with one variable in common (Steiger’s Z test).

Moodie, C. A., Suri, G., Goerlitz, D. S., Mateen, M. A., Sheppes, G., McRae, K., Lakhan-Pal, S., Thiruchselvam, R., & Gross, J. J. (2020). The neural bases of cognitive emotion regulation: The roles of strategy and intensity. *Cognitive, Affective and Behavioral Neuroscience*. https://doi.org/10.3758/s13415-020-00775-8

Ochsner, K. N., Silvers, J. A., & Buhle, J. T. (2012). Functional imaging studies of emotion regulation: a synthetic review and evolving model of the cognitive control of emotion. *Annals of the New York Academy of Sciences*. https://doi.org/10.1111/j.1749-6632.2012.06751.x

Phelps, E. A. (2006). Emotion and Cognition: Insights from Studies of the Human Amygdala. *Annual Review of Psychology*. https://doi.org/10.1146/annurev.psych.56.091103.070234

Pierce, J. E., James Blair, R. R., Clark, K. R., & Neta, M. (2021). Reappraisal-related downregulation of amygdala BOLD activation occurs only during the late trial window. *Cognitive, Afective, & Behavioral Neuroscience*, *1*, 3. https://doi.org/10.3758/s13415-021-00980-z

Poole, J. C., Dobson, K. S., & Pusch, D. (2017). Anxiety among adults with a history of childhood adversity: Psychological resilience moderates the indirect effect of emotion dysregulation. *Journal of Affective Disorders*. https://doi.org/10.1016/j.jad.2017.03.047

Price, J. L., & Drevets, W. C. (2010). Neurocircuitry of mood disorders. In *Neuropsychopharmacology*. https://doi.org/10.1038/npp.2009.104

Sabatinelli, D., Bradley, M. M., Fitzsimmons, J. R., & Lang, P. J. (2005). Parallel amygdala and inferotemporal activation reflect emotional intensity and fear relevance. *NeuroImage*. https://doi.org/10.1016/j.neuroimage.2004.12.015

Seligowski, A. V., Lee, D. J., Bardeen, J. R., & Orcutt, H. K. (2015). Emotion Regulation and Posttraumatic Stress Symptoms: A Meta-Analysis. In *Cognitive Behaviour Therapy*. https://doi.org/10.1080/16506073.2014.980753

Shafir, R., Schwartz, N., Blechert, J., & Sheppes, G. (2015). Emotional intensity influences pre-implementation and implementation of distraction and reappraisal. *Social Cognitive and Affective Neuroscience*. https://doi.org/10.1093/scan/nsv022

Sheldon, O. J., Dunning, D., & Ames, D. R. (2014). Emotionally Unskilled , Unaware , and Uninterested in Learning More : Reactions to Feedback About Deficits in Emotional Intelligence. *Journal of Applied Psychology*, *99*(1), 125–137. https://doi.org/10.1037/a0034138

Sheppes, G. (2014). Emotion regulation choice: Theory and findings. *Handbook of Emotion Regulation (2nd Edition)*.

Sheppes, G., & Gross, J. J. (2011). Is Timing everything? Temporal considerations in emotion regulation. *Personality and Social Psychology Review*. https://doi.org/10.1177/1088868310395778

Sheppes, G., & Meiran, N. (2007). Better late than never? on the dynamics of online regulation of sadness using distraction and cognitive reappraisal. *Personality and Social Psychology Bulletin*. https://doi.org/10.1177/0146167207305537

Sheppes, G., Scheibe, S., Suri, G., & Gross, J. J. (2011). Emotion-regulation choice. *Psychological Science*. https://doi.org/10.1177/0956797611418350

Sheppes, G., Scheibe, S., Suri, G., Radu, P., Blechert, J., & Gross, J. J. (2014). Emotion Regulation Choice: A Conceptual Framework and Supporting Evidence. *Journal of Experimental Psychology. General*. https://doi.org/10.1037/a0030831

1. **The codes of the pictures used**: High Intensity – IAPS (2053, 2352.2, 2730, 2981, 3000, 3005.1, 3010, 3015, 3030, 3053, 3060, 3062, 3064, 3068, 3069, 3071, 3080, 3100, 3101, 3102, 3110, 3120, 3130, 3140, 3150, 3168, 3170, 3180, 3185, 3261, 3266, 3301, 3400, 3530, 3550, 6212, 6230, 6313, 6350, 6360, 6510, 6520, 6540, 6838, 9040, 9050, 9181, 9183, 9250, 9252, 9253, 9265, 9300, 9400, 9405, 9410, 9412, 9413, 9420, 9433, 9500, 9560, 9570, 9571, 9600, 9635.1, 9908, 9910, 9911); EmoPicS (209, 210, 212, 224, 231, 232, 233, 234, 235, 236, 237, 238, 240, 241, 242, 243, 244, 245, 246, 247, 248, 249, 250, 251, 252); Stanford Psychophysiology lab (H01, H02, H03, H04, H05, H06, H07, H10, H12, H13, H14, H15, H16, H17, H18, H19, H21, H22, H23, H24, H27, H29, H30, H32, H35, H36, H37, H38,H39); GAPED (H1,H3,H4,H6,H9). Low Intensity – IAPS (1301, 2100, 2120, 2130, 2205, 2278, 2312, 2399, 2490, 2682, 2691, 2692, 2694, 2700, 2710, 2718, 2722, 2753, 2795, 2799, 2800, 2900.1, 3160, 3216, 3220, 3230, 3280, 3350, 6010, 6190, 6211, 6260, 6300, 6312, 6315, 6550, 6560, 6561, 6562, 6570.1, 6825, 6830, 6831, 6836, 6840, 7092, 7135, 7360, 7361, 7380, 7520, 7521, 8231, 9001, 9008, 9010, 9031, 9041, 9045, 9046, 9101, 9102, 9110, 9120, 9145, 9160, 9171, 9180, 9182, 9186, 9220, 9280, 9290, 9341, 9342, 9373, 9402, 9404, 9415, 9421, 9440, 9445, 9470, 9594, 9800, 9920); EmoPicS (207, 208, 214, 219, 220, 222, 226, 227, 228, 239); SPL (L02, L04, L05, L07, L09, L10, L12, L14, L16, L17, L18, L19, L20, L21, L22, L23, L24, L26, L28, L30, L31, L32, L33, L34); GAPED (L1, L2, L3, L4, L5, L6, L7, L9). Neutral: IAPS (7595, 7950, 7030, 7205, 7504, 7010, 5500, 7002, 7038, 7217, 7495, 7035, 7000, 7705, 7006, 7234, 7050, 7100); GAPED (N1, N2, N3, N4). [↑](#footnote-ref-1)
